# Cannabinoids and Multiple Sclerosis: A Critical Analysis of Therapeutic Potentials and Safety Concerns

**DOI:** 10.3390/pharmaceutics15041151

**Published:** 2023-04-05

**Authors:** Roua A. Nouh, Ahmed Kamal, Anwar Abdelnaser

**Affiliations:** 1 Department of Chemistry, School of Sciences and Engineering, The American University in Cairo, New Cairo 11835, Egypt; 2 Biochemistry Department, Faculty of Science, Suez University, P.O. Box 43518, Suez 43533, Egypt; 3 Institute of Global Health and Human Ecology, School of Sciences and Engineering, The American University in Cairo, P.O. Box 74, New Cairo 11835, Egypt

**Keywords:** multiple sclerosis, central nervous system, autoimmune disease, cannabinoids, tetrahydrocannabinol, *cannabis*, endocannabinoid system, acetylcholine, experimental autoimmune encephalomyelitis

## Abstract

Multiple sclerosis (MS) is a complicated condition in which the immune system attacks myelinated axons in the central nervous system (CNS), destroying both myelin and axons to varying degrees. Several environmental, genetic, and epigenetic factors influence the risk of developing the disease and how well it responds to treatment. Cannabinoids have recently sparked renewed interest in their therapeutic applications, with growing evidence for their role in symptom control in MS. Cannabinoids exert their roles through the endogenous cannabinoid (ECB) system, with some reports shedding light on the molecular biology of this system and lending credence to some anecdotal medical claims. The double nature of cannabinoids, which cause both positive and negative effects, comes from their actions on the same receptor. Several mechanisms have been adopted to evade this effect. However, there are still numerous limitations to using cannabinoids to treat MS patients. In this review, we will explore and discuss the molecular effect of cannabinoids on the ECB system, the various factors that affect the response to cannabinoids in the body, including the role of gene polymorphism and its relation to dosage, assessing the positive over the adverse effects of cannabinoids in MS, and finally, exploring the possible functional mechanism of cannabinoids in MS and the current and future progress of cannabinoid therapeutics.

## 1. Introduction

Multiple sclerosis (MS) is a neurodegenerative condition that can cause paralysis, demyelination, and harm to neuronal axons [1]. Inflammation and myelin sheath degeneration are the hallmarks of MS that lead to lesions, which have been found in the white matter of the brain stem, optic nerve, and spinal cord [2]. The myelin sheath is destroyed via the immune system, which begins to perceive its constituent parts as foreign components. Infiltration of the immune cells is thought to cause plaque formation, which initiates disease symptoms through releasing cytokines and inflammatory mediators that cause inflammation, myelin damage, oligodendrocyte loss, neuronal function loss, and eventually axonal degeneration [3,4]. MS’s signs and symptoms depend on where the lesions are in the brain or spinal cord [5]. MS has three stages: pre-clinical, relapsing/remitting, and progressive clinical. Depending on the severity of the disease, the symptoms can differ from person to person. They can result in short-term, long-term, or even permanent losses due to disrupted signal transmission. The complete remission and the treatment of progressive forms of MS remain controversial and a medical challenge, even though numerous medications have been developed for the disease [6]. The drugs used to manage MS are classified into two main groups: disease-modifying agents and symptomatic treatment [7]. Symptomatic treatment aims to decrease the symptoms, but it is limited by its toxicity [8]. Recently, the therapeutic uses of cannabinoids as a symptomatic treatment has been gaining popularity, with many trials and patients, who believe that it may help with the management and control of symptoms in MS. Despite the overwhelming evidence supporting the use of cannabinoids in the treatment of MS, there is still a lack of knowledge regarding the precise effects of cannabinoids due to patient variability. In this review, we will explore and discuss the molecular effect of cannabinoids on the ECB system, the various factors that affect the response to cannabinoids in the body, including the role of gene polymorphism and its relation to dosage, assessing the positive over the adverse effects of cannabinoids in MS, and finally, exploring the possible functional mechanism of cannabinoids in MS and the current and future progress of cannabinoid therapeutics.

## 2. Cannabinoids and the Endocannabinoid System

*Cannabis sativa* is the main species of the complex plant known as Cannabis, a member of the *Cannabacea* family [9]. The *Cannabis sativa* plant, known as hemp, has been known as a psychoactive substance for over 4000 years due to its hallucinogenic characteristics. Cannabis is also known as marijuana, among other local names [10]. 

More than sixty physiologically active chemical substances, known as cannabinoids, can be created either naturally (phytocannabinoids), by animals (endocannabinoids), or artificially (synthetic cannabinoids) [11]. The cannabis plant contains more than 100 phytocannabinoids, including the 2 most significant ones, which are Δ⁹-tetrahydrocannabinol (THC) and cannabidiol (CBD). Δ⁹-THC, commonly referred to as THC unless stated otherwise, is believed to be the main psychoactive compound found in cannabis [12]. Unlike the phytocannabinoids found in cannabis, all endocannabinoids are derived from Arachidonic acid and, therefore, have different chemical structures. At present, there are five known endocannabinoids, including N-Arachidonoyl Ethanolamide (also called Anandamide), 2-Arachidonoyl Glycerol, 2-Arachidonoyl Glyceryl Ether (Noladin ether), O-Arachidonoyl Ethanolamine (Virodhamine), and N-Arachidonoyl Dopamine (NADA). Among these endocannabinoids, Anandamide and 2-Arachidonoyl glycerol are considered the most significant [13]. The Chinese pharmacopeia has referred to cannabinoids as a therapeutic agent since 400 AD [14]. Since 2015, there has been an upsurge in cannabinoid products and delivery systems, and an increase in the number of nations legalizing cannabinoids, possibly due to greater public awareness of the drug’s potential for medical use [15]. 

**Figure 1:**
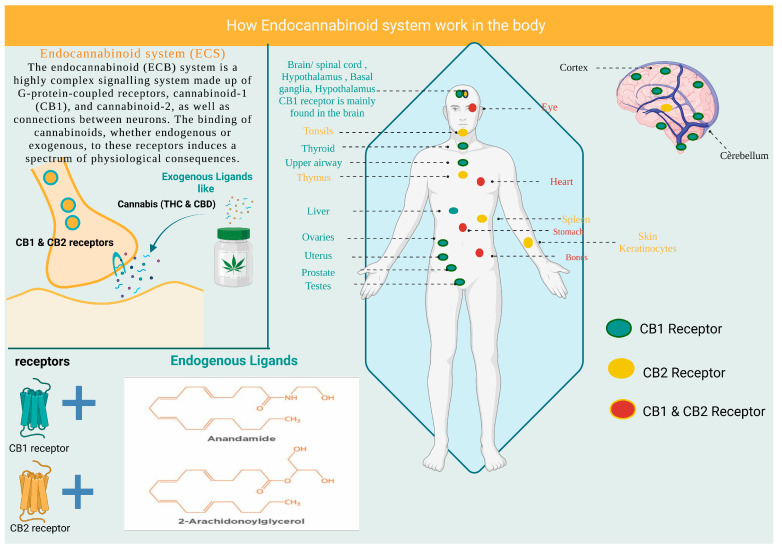
Illustration of the endocannabinoid system and distribution of CB1 receptors. CB1: cannabinoid-1 receptor, CB2: cannabinoid-2 receptor, THC: tetrahydrocannabinol, CBD: cannabinoid.

The endocannabinoid (ECB) system is a highly intricate signaling system composed of neuronal connections, endocannabinoid neurotransmitters, and G-protein-coupled receptors cannabinoid-1 (CB1) and cannabinoid-2 (CB2). The binding of cannabinoids, whether endogenous or exogenous, to these receptors, produces a range of physiological effects [16]. The ECB receptors are dispersed centrally and peripherally, as shown in Figure 1 [17]. 

## 3. Molecular Effect of Cannabinoids on the Central Nervous System

The impact of cannabinoids on the molecular level in the brain is one of the crucial elements that need to be thoroughly explained. 

### 3.1. Role of CB1 and CB2 Receptors

**Figure 2:**
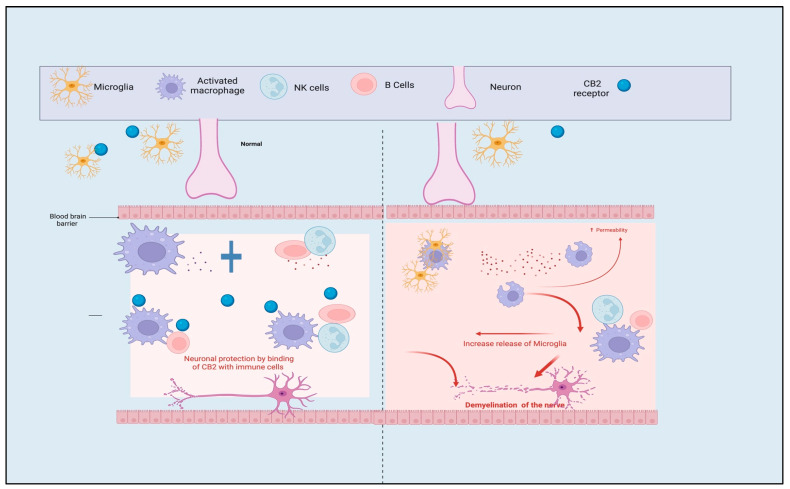
The physiological function of the CB2 receptor during leukocyte transmigration and inflammation, the transport of immune cells across the BBB is restricted by CB2 receptors. CB2: cannabinoid-2 receptor, NK: natural killer cells, B cells: B lymphocytes cells.

CB1 receptors, which are primarily present in nerve terminals, are responsible for the inhibition of the release of neurotransmitters. On the other hand, CB2 receptors are expressed mainly by immune cells, and among their roles is the regulation of cytokine production and immune cell movement both inside and outside of the central nervous system. Additionally, recent research has shown that some neurons and blood arteries in the brain have CB2 receptors [18,19]. The ECB system mediates neuroprotective actions via glutamatergic neurons, while inflammatory responses are regulated via GABAergic neurons and astrocytes. Many neuronal disorders, including age-related neurodegeneration, are linked to changes and dysfunction of the ECB system [19]. Age-related cognitive decline is associated with decreased CB1 receptor expression in the hippocampus, and CB1 receptor deletion from GABAergic hippocampal neurons results in neuronal death and elevated inflammation [20]. Moreover, changes in CB1 receptor signaling that are unique to glutamatergic neurons may impact age-related cognitive decline and decreased synaptic integrity and plasticity. Cannabinoids that signal through the CB1 receptor cause synaptic plasticity, cell migration, and neuronal growth, whereas cannabinoids that signal through the CB2 receptor are associated with mechanisms that stop, slow down, and repair damage caused by inflammation, as illustrated in Figure 2 [21].

### 3.2. Molecular Effect of Cannabinoids and ECB System in the Hippocampus

Higher-order brain activities rely on coordinating various delicately balanced systems like an orchestra, which executes the rhythms that shape our cognitive processes, affect our behavior, and create our memory. The hippocampus has drawn more attention than any other brain region. Consequently, it is an essential component of mnemonic systems in various species, including humans. Additionally, several studies have suggested that the hippocampus is a center for conscious and unconscious experience [22,23]. It is essential to explore the molecular mechanisms controlling hippocampus circuitries to understand the comprehend neural processes. Molecular effectors are seen as CNS modulators indicating that their actions impact cognition and behavior. The hippocampus plays a role in cognitive functions such as memory, learning, and sensory integration [24,25].

Additionally, it contains a lot of CB1 receptors, which are crucial for controlling pathophysiological processes [25,26]. The CB1 receptors are found mainly in the hippocampus’ GABAergic neurons and are also present in glutamatergic neurons, astrocytes, and subcellular compartments [27,28]. Cannabinoid signaling regulates the release of cholinergic and dopaminergic neurotransmitters in addition to the typical excitatory/inhibitory transmission regulation by CB1 receptors [29,30]. Acetylcholine (ACh) and GABA are released at mixed GABAergic synapses established explicitly by the cholinergic terminals in the hippocampus [31]. In this situation, regulating dopaminergic and cholinergic transmission in the hippocampus under CB1 receptor-dependent may control cognitive and affective processes.

Interestingly, cannabinoid antagonist use has been linked to improved cognitive abilities. This can be explained by increased acetylcholine levels brought on by cholinergic disinhibition in the hippocampus. In ACh neurotransmission, Δ^9^-tetrahydrocannabinol (Δ^9^-THC) exhibits a biphasic, dose-dependent impact. Because ACh levels are governed by dopamine receptor activation, only high dosages of THC can reduce their levels. As a result, while septal D1 receptor signaling increases ACh efflux, hippocampal D2 receptor activation induces ACh suppression triggered by large dosages of THC [32]. Dopaminergic terminal activity in the ventral tegmental region is increased when endogenous or exogenous cannabinoids activate the intrahippocampal CB1 receptor. These systems must be appropriately controlled for the hippocampus to function at its peak, particularly during stressful situations when alterations in neurotransmitter levels may cause maladaptive changes connected to neuropsychiatric disorders [33]. This molecular mechanism emphasizes the critical role of the endocannabinoid system in the progression or management of neurodegenerative disease, implying the possibility of developing treatments that can cure MS as well as many other neurodegenerative disorders.

### 3.3. Effect of Cannabinoids on Mitochondria and Metabolic Pathway

The brain accounts for only 2% of total body weight in mammals but utilizes up to 20% of the body’s energy production [34,35]. Mitochondria are crucial elements of eukaryotic cell functions [34,36,37]. The role of neuronal energetics in brain physiology and pathology is the subject of extensive research. The molecular mechanisms linking mitochondrial activity to brain functions remain unclear. CB1 receptors have recently been discovered in the mitochondria of hippocampus astroglia cells [27], although it is still unclear how they might affect astrocytic activity and possibly modify brain networks. The activation of the mitochondrial CB1 receptor (mtCB1) has been linked to alterations in bioenergetics and mitochondrial respiration that influence hippocampus synaptic transmission and, in turn, memory consolidation [38,39]. By turning on a subgroup of mitochondrial G proteins called Gi proteins, the mtCB1 receptor slows down mitochondrial respiration. Proteins involved in oxidative phosphorylation are not phosphorylated as much as before as a result of this activation, which also decreases protein kinase A (PKA) activity in the mitochondria. Since synaptic transmission requires a significant amount of energy, any disruptions in mitochondrial respiration directly impact synaptic activity. Long-term memory is also disturbed when the mtCB1 receptor is activated because it decreases ATP and prevents excitatory synaptic transmission in the CA1-CA3 circuit [39]. A recent study connected the disturbance of glucose metabolism and lactate generation to the activation of astroglia mtCB1 receptors [28]. THC administration results in decreased mitochondrial protein phosphorylation, which changes reactive oxygen species (ROS) levels, causing the transcription factor hypoxia-inducible factor 1 to be downregulated (HIF-1). Since the HIF-1 pathway promotes glycolysis, inhibiting it has a detrimental impact on glucose metabolism and lactate generation.

## 4. Pharmacokinetics and Pharmacodynamics Characteristics of Cannabinoids

Cannabinoids have been proven to have anti-inflammatory, antiviral, and anticancer characteristics, according to studies on the pharmacodynamics of cannabinoids [40]. Recognizing the pharmacokinetics and pharmacodynamics characteristics of cannabinoids is crucial to understanding the effects of drugs on the body. Most of the pharmacodynamics and pharmacokinetic data were obtained through studies on cannabinoid users or healthy volunteers. Several factors impact the pharmacokinetics of cannabinoids, such as prior cannabinoid usage, pharmacogenetics, body size, disease status, food, and microbiome. The dosage form of cannabinoids and the route of administration impact the pharmacokinetics parameters of cannabinoids, as shown in Table 1 [41]. The biodistribution of cannabinoids can be affected by their lipophilicity, where THC has a high distribution volume (5.7–10 L/kg) due to its high lipophilicity. The distribution of CBD is also affected by its lipophilicity and has a high volume of distribution, and it can rapidly penetrate the brain, adipose tissue, and other organs [42]. Another factor that may increase the volume of distribution is the chronic regular administration of cannabinoids which precipitates tissue accumulation over time [42,43]. The liver predominantly metabolizes cannabinoids. A tiny amount of extra-hepatic metabolism exists in tissues such as the brain, intestines, and lungs [44]. The cytochrome P450 (CYP 450) enzymes, specifically CYP2C9, CYP2C19, and CYP3A4, metabolize THC in the liver. Decarboxylation, epoxidation, and oxidation are all steps in THC metabolism that occur before conjugation. The two primary THC metabolites are Δ^11^-hydroxy-THC (Δ^11^-OH-THC) and Δ^11^-carboxy-THC (Δ^11^- COOH-THC), which are produced when THC is hydroxylated and oxidized, respectively. Tissues that express CYP 450 participate in the extra-hepatic metabolism of THC [45].

Additionally, CBD is metabolized in the liver via the CYP 450 isoenzymes CYP2C19 and CYP3A4, where it is then subjected to different metabolic processes before being excreted [46]. The route of elimination is also a pharmacokinetic characteristic in which THC and CBD metabolites are eliminated through the urine, feces, and, to a lesser extent, bile. The elimination rate is influenced by several variables, including the type of dosage form and the patient’s characteristics [40]. Until now, no clear guideline involves specific instruction about the required dose of cannabinoids. Consequently, there is a need to conduct studies on the kinetics of the drug with different patient populations and diseases, specifically the MS population. 

**Table 1:** Effect of various dosage forms on pharmacokinetics parameters (PKM) of cannabinoids.

	PKM	Absorption	Bioavailability	Peak Plasma Concentration	Duration of Action	Advantages	Disadvantages
Dosage Form							
Inhalation		High absorption = 10–60%	THC = 2–65%, CBD = 6–31%	Peak plasma concentrations of THC and CBD are reached quickly, within 3–10 min.	1–4 h	Suitable bioavailability and rapid onset of action.	Variability between patients based on lung function.
Oral		Low absorption = 2–14%	THC = 5–10%, CBD = 6–20% Due to the effect of 1st pass metabolism.	Achieve peak concentration (60–120 min).	6 h	It provides a sufficient duration of action.	Delayed onset of action.
Topical		Irregular absorption	Skin barriers hinder bioavailability due to the lipophilic nature of the drug.	Steady-state condition is achieved within = 17 h.	THC = 14 h, CBD = 72 h	Reduction in the side effects associated with systemic administration of the drugs.	Poor bioavailability due to skin barrier.
Systemic intravenous		High absorption rate	High bioavailability like inhaled dosage form.	Within 10 min.	4 h	Rapid action and high bioavailability.	Require an aqueous vehicle due to poor water solubility.
Ref.		[15,43,44]	[15,47,48]	[15,49]	[49,50]	[50,51]	[15,52]

## 5. Pharmacogenetics and Cannabinoids

Gene polymorphisms implicated in drug action, metabolism, and transport in the body may be the reason for response variation in MS patients responding to cannabinoid therapy. The most recent data on gene polymorphisms influencing cannabinoids transport, activity, and metabolism will be discussed here. The genetic alteration that can affect the response to cannabinoid therapy can be divided into variations of genes that code for cannabinoid receptors or those that code for metabolizing enzymes.

### 5.1. Variation of Genes That Code for Cannabinoid Receptors

The majority of studies on the effects of cannabinoids have focused only on a particular class of receptors that cannabinoids act on. There are more than one or two cannabinoid receptors with specific effects on the body’s function that require additional research, especially in the population of MS. The ECB system has various types of receptors, which may impact the activity of ligands in many physiological processes, and scientists have demonstrated in recent years that this mechanism is far more complex [53]. Variations in the expression of these receptors will affect cannabinoids and their effects on the body, as shown in Table 2.

#### 5.1.1. Polymorphism of Cannabinoid Receptors CB1 and CB2

The discovery and cloning of the first and second cannabinoid receptors (CB1 and CB2) in 1990 and 1993 significantly raised our knowledge about cannabinoids. The CNR1 gene, which codes for the CB1 receptor, is found on human chromosome 6q14–15 and has four exons, the biggest of which is most frequently expressed in brain tissue [60]. The CB1 receptor is characterized by evolutionary conservatism. The CB receptor’s activation increases hunger and has sedative, analgesic, and antiemetic properties [61]. Contradictory findings were found in research linking the CNR1 gene’s single nucleotide polymorphism (SNP) with marijuana addiction, which also included the trinucleotide repeat locus (AAT) and the insertion-deletion (-3180T) polymorphism [54]. In 206 non-Hispanic Caucasians (92 subjects and 114 controls), Comings et al. found a link between (AAT) >5 repeats and drug dependency [62]. However, most trinucleotide repeat (AAT)n studies had contradictory findings [63,64]. Moreover, the Hartman Group found that in case-control samples, the CNR1 gene variant rs1049353 was associated with symptoms of cannabinoid dependency [65]. CNR2, which codes for CB2, is the second gene chosen. There are not many descriptions in the literature of studies on the CNR2 gene and addiction. Mutant CB2 receptors were then transfected into HEK293 cells, according to Carrasquer et al. This study showed that the CB2 polymorphic receptors might bind cannabinoid ligands at locations 63 and 316 and mediate signal transmission, which may help explain the etiology of some disorders [66].

#### 5.1.2. Transient Receptor Potential Cation Channel Subfamily V Member 1 (TRPV1)

The transient receptor potential cation channel subfamily V member 1, also known as TRPV-1, is a ligand-gated, non-selective ion channel discovered as another receptor target for cannabinoids. TRPV-1 has been shown to express itself in a wide range of cells, including those of the immune system, the central and peripheral nervous systems, endothelium and epithelial cells, keratinocytes, and smooth muscle cells [11]. On primary afferent nerve cells, there is a more significant co-localization of cannabinoid receptors and TRPV-1. It has been demonstrated that the endocannabinoid AEA acts as an endo vanilloid by stimulating the TRPV-1 receptor, which mediates downstream signaling [67].

Until now, no association studies have shown a connection between the presence of TRPV1 gene polymorphism and marijuana addiction. Two well-known receptors, CB1 and CB2, are the focus of most studies on the functioning of the ECB system. CBD’s two enantiomeric variants have little or no affinity for the CB1 and CB2 receptors. Therefore, it is conceivable that the effectiveness of CBD is linked to its ability to reduce the activity of FAAH and increase anandamide levels (AEA). This would suggest that elevated MDR1 mRNA expression following CBD exposure depends on AEA’s simultaneous activation of CB2 and TRPV1 [53]. 

### 5.2. Variation in Genes That Code for Metabolizing Enzymes

The metabolism of cannabinoids is enzyme-dependent [68]. Oxidation, decarboxylation, and conjugation with glucuronic acid are the primary mechanisms used in metabolizing Δ^9^-THC. There is no doubt that environmental factors such as drug availability and social conditioning impact cannabinoid usage, but more research is required to understand the genetic effect on cannabinoids fully. The CYP gene family consists of 58 pseudogenes and 57 putatively functional genes in humans. Alterations in or inactivation of enzyme activity can result from CYP gene polymorphism and mutation. Cytochrome P450 (CYP, EC1.14.14.1) superfamily enzymes, specifically CYP3A4, are considered a significant metabolic pathway for THC, CBD, and CYP2C9 encoded by CYP2C9 and CYP3A4 genes are predicted to play an essential role in the primary metabolism of THC. The highly polymorphic CYP2D6 gene, a member of the CYP superfamily, has been implicated in discovering a phase I enzyme with over 91 distinct alleles [69]. The most prevalent CYP2D6 variants are *3, *4, and *5, with phenotypes indicative of poor metabolizers and decreased or absent enzyme activity. There is a wide range of CYP2D6 activity in people with genetic polymorphisms in the CYP2D6 gene. Moreover, xenobiotics and endobiotics are glucuronidated by a superfamily of enzymes known as UDP-Glucuronosyltransferases (UGTs).

The UDP-glucuronosyltransferases (UGTs) superfamily of enzymes is a key player in detoxifying and eliminating both endogenous and exogenous substances, such as cannabinoids and their metabolite, through the process of glucuronidation. Of all phase II drug metabolites, 35% or less are glucuronides [70]. The UGT families consist of UGT1, UGT2, UGT3, and UGT8 [71]. Additionally, recent research has shown new polymorphisms connected to the various enzyme alleles, as illustrated in Table 3. In addition to the disease-modifying therapies that treat critical MS, dose requirements for several commonly used drugs with a narrow therapeutic range may differ by more than 20-fold depending on genotype or enzyme expression status [72].

## 6. Adverse Effects of Cannabinoids

Cannabinoids are often dismissed by the general public as a safe substance, oblivious to any potential long-term health issues [83,84,85]. Comparing cannabinoid users with non-users in the general population reveals that cannabinoids negatively impact cognition [86]. There is growing evidence that acute cannabinoid use is linked to other neurocognitive decision-making deficits, including processing speed, sustained attention, verbal fluency, and executive functioning [87,88,89,90,91,92,93,94]. Chronic cannabinoid usage by teenagers and young adults over time affects cognitive abilities in several domains, including learning, memory, attention, decision-making, executive functioning, and psychomotor speed [85,95,96,97,98]. However, one systematic review investigated the adverse effects of cannabinoids in progressive MS patients who never used cannabinoids. The results indicated potential improvements in cognition with medicinal cannabinoids for MS patients. However, this effect is only for short-time use. It contradicts the findings that chronic use of whole-plant cannabinoids resulted in impairments in memory, attention, and executive and visuospatial function [99]. More research is required for the detailed investigation of the adverse effects of cannabinoid use both in the short and the long term.

A recent review on the adverse effects of cannabinoids has discussed not only the implications of cannabinoids on mental functions, psychiatric conditions, and cognitive and CNS alterations but also their effects on the respiratory system, the immune system, the reproductive system, and the cardiovascular system [100]. Therefore, the use of cannabinoids in the medical field must be conducted with great caution to benefit from their potential benefits while avoiding the possible risks.

## 7. Cannabinoids and the Management of Multiple Sclerosis

Earlier studies using an in vitro model of MS, the Experimental Autoimmune Encephalomyelitis (EAE) model, have shown that cannabinoids are highly effective in treating MS [101]. The cannabinoid receptor agonists Tetrahydrocannabinol, Δ^8^- and Δ^9^-THC appear to lessen the symptoms of EAE and significantly reduce the immune response in animals. Several studies have shown that clinical signs of EAE, such as tail flaccidity and generalized atonia, were delayed in onset and diminished in severity after these pharmacological treatments [102,103]. Additionally, one of those studies found that Δ^9^-THC reduced the histological evidence of EAE inflammation in the spinal cord of rats and guinea pigs [102]. Interestingly, dexanabinol, one of the synthetic cannabinoid ligands that lack the potential to act through CB1 or CB2 receptors, has also been shown to lessen EAE symptoms in rats [104]. 

Numerous clinical studies indicate that the pain and spasticity caused by MS and spinal cord injuries may be effectively reduced via cannabinoids, whose potential to reduce the signs and symptoms of MS and spinal cord damage is consistent with some of the drug’s conventional medical uses. Clinical investigations have shown that cannabinoid receptor agonists help lessen some of the MS-specific signs and symptoms, and these clinical trials are depicted in great detail in Table 4.

The promising effects of cannabinoids in managing various effects of MS are something that all of the research from 1996 to 2020 has in common. For example, neuropathic pain, one of the significant MS symptoms, has been demonstrated to be reduced by using cannabinoids. Several clinical studies involving various cannabinoids and dosage forms produced promising outcomes for the prevention and/or treatment of neuropathic pain. However, some clinical trials used personal anecdotes gleaned through distributing questionnaires to MS patients using cannabinoids for self-medication. The first survey used responses from 57 male and 55 female patients [105]. The results pointed out that more than 90% of these individuals who were experiencing MS symptoms, such as spasticity at the start of sleep, muscle discomfort, pain in the legs at night, tremor in the arms or head, and depression, have reported relief after using cannabinoids. Despite these findings, there is conflicting evidence about the effectiveness of cannabinoids in treating MS and spinal cord damage. It is currently unknown if cannabinoid receptor agonists prevent MS from progressing in patients with this condition or if they only lower the intensity of specific signs and symptoms. There is still some debate in the literature about the relative importance of CB1 and CB2 receptor activation and the causes of variations in response to cannabinoids. Many factors, such as dosage form, genetic makeup, or the molecular effects of cannabinoids at different doses, contribute to patient response variation.

**Table 4:** Various clinical trials on the use of cannabinoids with MS patients.

Drug (Active Constituent)	Dosage Form	Experimental Design	Outcomes	Ref.
THC (5–10 mg)	Oral	Double-blind study	Decrease muscle spasms and enhance the walking ability of the patient.	[106]
Nabilone (1 mg), a synthetic THC mimic	Oral	Open-Label study	Dimension in the pain with MS patients.	[107]
THC (10 mg oral or 15 mg rectal)	Oral/rectal	Open-Label study	Enhancement of walking ability, passive mobility, and dimension in the pain with young MS patients.	[108]
THC (7.5 mg)	Oral	Placebo-controlled, double-blind study	Improved muscle spasm perception.	[109]
Sativex, a THC-CBD combination (2.7 mg: 2.5 mg)	Oromucosal	Controlled double-blind, randomized study	It has an analgesic effect in addition to enhancing of quality of life of the patient (e.g., sleep improvement).	[110]
Tobacco and cannabis resin-containing smoking	Inhalation	Placebo-controlled trial	Decrease in the Nystagmus amplitude and enhancement of visual ability.	[111]
Dronabinol (>25 mg), a synthetic THC mimic	Oral	Randomized, double-blind controlled study	Significant analgesic effect.	[8]
THC (5–15 mg)	Oral	The single-blind study was a placebo-controlled trial	Enhancement of the patient’s ability in handwriting and a significant decrease in spasticity and tremors.	[112]
THC (5–10 mg)	Oral	Double-blind study	Improvement in tremors and ataxia with MS patient.	[113]
Nabilone (1 mg), a synthetic THC mimic	Oral	Double-blind controlled trials	Reduction in muscle spasms and tremors.	[114]
THC (10–15 mg oral or 2.5–5 mg rectal)	Oral/rectal	Open-Label study	Analgesic effect with MS patient.	[108]
Sativex, a THC-CBD combination (2.7 mg: 2.5 mg)	Oromucosal	Double-blind controlled trials	Improvement in muscle spasms in MS patients.	[115]
THC (1 mg)	Oral	Open-Label study	Have an analgesic effect on patients.	[107]
Sativex, a THC-CBD combination (2.7 mg: 2.5 mg)	Oromucosal	Double-blind controlled randomized trial	Improving the resistant MS spasticity more effectively and clinically.	[116]
Sativex, a THC-CBD combination (2.7 mg: 2.5 mg)	Oromucosal	Observational, prospective controlled trial	Improvement in the symptoms of MS in resistant patients.	[117]
Sativex, a THC-CBD combination (2.7 mg: 2.5 mg)	Oromucosal	Double-blind controlled trial	Improvement in the clinical states of MS patients.	[118]
Sativex, a THC-CBD combination (2.7 mg: 2.5 mg)	Oromucosal	Randomized controlled study	Decrees the neuropathic pain associated with MS patients.	[119]
Sativex, a THC-CBD combination (2.7 mg: 2.5 mg)	Oromucosal	Open-Label study	This study has proven the immunomodulatory effect of cannabinoids by detecting the gene expression of immune-related pathways.	[120]
Sativex, a THC-CBD combination (2.7 mg: 2.5 mg)	Oromucosal	Randomized controlled trial	A significant reduction in the pain associated with MS.	[121]
Sativex, a THC-CBD combination (2.7 mg: 2.5 mg)	Oromucosal	Controlled retrospective study	It demonstrates an efficient and safe reduction in muscle spasms.	[122]

## 8. Functions of Cannabinoids in MS

Animal models have been used to investigate the possible function of cannabinoids in preventing the progression of MS and providing neuroprotection. One of the most accepted mechanisms of action is immunosuppression. This effect was revealed in an in vivo study using an EAE model of mice and a daily dose of CBD, which showed dimensions in the T cell infiltration and neuroinflammation in the brain and spinal cord’s white matter pathways [123]. Another similar study used CBD alone or in combination with THC. In both techniques, there was a reduction in the proliferation and number of T cells, which impacted and reduced the degree of demyelination of neurons [124]. 

In vitro studies also supported the immunosuppression mechanism as the most dominant pathway of protection in MS. Two earlier studies used THC on either animal or human cell cultures, and the results showed inhibition or reduction of T cells’ proliferation [125,126]. A recent study has also demonstrated that THC decreases the number of natural killer cells (NK) [127]. Recent studies extended to the use of CBD in cell cultures. A study by Yang et al. investigated the use of CBD on T cells and showed that the proinflammatory phenotype of T cells was reversed [128]. It is also noteworthy that the effect of cannabinoids is not exclusive to T cells, as demonstrated by a study by Kozela et al., which showed that the use of CBD negatively impacted both T cells and B cells [129].

## 9. The Growing Field of Cannabinoid Therapeutics

Several clinical studies have been conducted on human individuals (Table 4). The results showed great promise for using cannabinoids in MS. Commercial cannabinoid formulations have advanced in recent years, but their use is still limited. The combined effects of THC and CBD can be observed in Sativex, one example of a commercial cannabinoid formulation. Many clinical trials have been conducted to evaluate the efficacy of Sativex as a supplemental therapy for patients with MS who have moderate to severe spasticity [116,130]. Nabiximols, a combination of CBD and Δ^9^-THC formulation, have also been used to manage spasticity associated with MS [131]. Neuropathic pain, a common symptom of MS that affects between 17% and 70% of patients, was also shown in a study to be reduced by Sativex. Additionally, Sativex is well tolerated by MS patients and has a low incidence of side effects [132].

Although the number of cannabinoids in formulations approved for the treatment of MS is still limited, several cannabinoids are used to treat a variety of other diseases and conditions, such as Nabilone for the management of Parkinson’s disease [133], Dronabinol, which is used to decrease anorexia, disturbed behavior [134], and nighttime agitation [135] in Alzheimer’s disease, and Cesamet for the treatment of chemotherapy-induced nausea in cancer patients [136]. All these indicate that the field of cannabinoid therapeutics is still in its infancy, but it will witness remarkable progress in the future.

## 10. Future Research on Cannabinoids 

It has been discovered that cannabinoids may bind to a variety of locations, including the transient receptor potential vanilloid subtype 1 (TRPV1), the G-protein-coupled receptor 55 (GPR55), and the cannabinoid CB1 and CB2 receptors [100]. Despite the accepted scientific fact that cannabinoids act through interactions with the receptors of the ECB system, which are CB1 and CB2 [16], the idea that a drug may interact with several proteins to exert its biological function has slowly gained acceptance. Therefore, a recent review by Zhu et al. discussed the recent updates on natural products and the different approaches for target identification via binding affinity experiments for target recognition and validation and the biological function verification of the significance of this binding. This review also pointed out single-cell multi-omics and pathway enrichment analysis for identifying gene regulatory networks and the full impact of drug interactions on system biology [137]. Future research on cannabinoids using this approach will enable us to understand further the potential benefits and safety risks of cannabinoids for several diseases and conditions.

## 11. Concluding Remarks and Perspectives

Multiple sclerosis (MS) is a neurodegenerative condition in which inflammation and myelin degeneration lead to lesions, which have been found in the white matter of the brain stem, optic nerve, and spinal cord [2]. MS’s signs and symptoms depend on where the lesions are in the brain or spinal cord [5]. Symptomatic treatment aims to decrease the symptoms, but it is limited by its toxicity [8]. More than sixty physiologically active chemical substances, known as cannabinoids, can be created either naturally (phytocannabinoids), by animals (endocannabinoids), or artificially (synthetic cannabinoids) [11]. The therapeutic use of cannabinoids as a symptomatic treatment for MS has recently grown in popularity, where they exert their function through the endocannabinoid (ECB) system, which is a complex signaling system that includes the G-protein-coupled receptors cannabinoid-1 (CB1) and cannabinoid-2 (CB2) [16].

Cannabinoids have been proven to have anti-inflammatory, antiviral, and anticancer characteristics, according to studies on the pharmacodynamics of cannabinoids [40]. However, the effects and responses of cannabinoids can vary among individuals due to genetic variations in cannabinoid receptors or metabolizing enzymes, as shown by different studies in Table 2. Therefore, cannabinoid treatment should be tailored to an individual’s genomic state rather than used indiscriminately. The potential benefits of cannabinoids must also be balanced with the associated risks, including adverse effects on mental, cognitive, and physical functions and the respiratory, immune, reproductive, and cardiovascular systems [100]. Therefore, the medical use of cannabinoids must be approached with caution.

Since the 1990s, the therapeutic use of cannabinoids in MS has been studied through in vitro experiments, in vivo pre-clinical studies on animals, clinical trials on human subjects, and patient questionnaires assessing symptom relief after self-medication with cannabinoids. All these studies showed the potential therapeutic benefits of cannabinoids in MS. Some of them advanced to produce commercial therapeutic formulations of cannabinoids such as Sativex, which is used as a supplemental therapy for patients with MS who have moderate to severe spasticity [116,130], and Nabiximols, which has also been used for the management of spasticity associated with MS [131]. However, despite extensive previous research, further studies are needed on cannabinoids to enhance their safety and efficacy in treating MS and other diseases.

## Figures and Tables

**Table 2:** Genetic variation and their relation to cannabinoids response.

Protein	Gene	Function	Variation	Effect	Ref.
CB1	CNR1	Receptor	63-9597T > C	Cannabinoids addiction	[54]
CB2	CNR2	Receptor	946C > T	The effect and the main function of the CB2 receptor are altered	[54]
FAAH protein	Fatty Acid Amide Hydrolase FAAH	Biotransformation	385C > A	It is associated with drug abuse	[55]
Catechol-O-methyltransferase enzyme	COMT	The regulation and inactivation of catecholamine neurotransmitters in the brain	472A > G	Modest controllable effect of cannabinoids consumption on executive functions	[56]
GABA	*GABRA2*	Receptor	231A > G	No significant effect on the drug dependence	[57]
Mu opioid receptor	*OPRM1*	Receptor	118A > G	No significant effect	[58]
*ErbB3, ErbB4*	*NRG1*	Promotes the growth, differentiation, and survival of a wide range of cell types	122-16329C > T	Associated with cannabinoids dependence	[59]

**Table 3:** Various alleles and their relation to the metabolizing enzyme activity.

Gene	Allele	Nucleotide Change	Effect	Ref.
*CYP2D6*	CYP2D6*3	A2549del	It will produce a protein with little or no function, which means increased activity of cannabinoids in the body.	[73]
*CYP2D6*	CYP2D6*4	G1846A	The activity of the enzyme is reduced, causing drug accumulation in the body.	[73]
*CYP2C9*	CYP2C9*2	c.430C > T	The activity of the enzyme is reduced, causing drug accumulation in the body.	[74]
*CYP2C9*	CYP2C9*3	c.1075A	The activity of the enzyme is reduced, causing drug accumulation in the body.	[74]
*CYP3A4*	CYP3A4*2	664 T-C	The activity of the enzyme is reduced, causing an increase in the drug’s half-life time.	[74]
*CYP3A4*	CYP3A4*11	1088 C-T	The activity of the enzyme is reduced, causing an increase in the drug’s half-life time.	[75]
*CYP3A4*	CYP3A4*12	1117 C-T	The activity of the enzyme is reduced, causing an increase in the drug’s half-life time.	[76]
*CYP3A4*	CYP3A4*17	566 T-C	Decrease in the enzyme activity, which increases the half-life of the drug.	[69]
*UGT1A*	UGT1A9*3a	98T-C	Reduction or inactivation of the enzyme.	[77]
*UGT1A*	UGT1A9*4	726 T-G	Reduction or inactivation of the enzyme.	[78]
*UGT1A*	UGT1A9* 5	766 G-A	Reduction or inactivation of the enzyme.	[79]
*UGT1A*	UGT1A1*3	1124 C-T	It will lead to the inactivation of the enzyme.	[80]
*UGT1A*	UGT1A1*10	1021C-T	Dimension in the enzyme activity.	[81]
*UGT1A*	UGT1A1*13	508-510del	Inactivation of the enzyme.	[82]

## Data Availability

All figures (Figure 1 and Figure 2) in this manuscript were constructed using Biorender (www.biorender.com). Additionally, a publication license was obtained from Biorender for the use of these figures in this manuscript, which allows for the reproduction and distribution of the figures.
